# The Binding Properties of Glycosylated and Non-Glycosylated Tim-3 Molecules on CD4^+^CD25^+^ T Cells

**DOI:** 10.4110/in.2009.9.2.58

**Published:** 2009-04-30

**Authors:** Mi Jin Lee, Yoo Mi Heo, Seung-Ho Hong, Kyongmin Kim, Sun Park

**Affiliations:** Department of Microbiology and Immunology, Ajou University School of Medicine, Suwon 442-721, Korea.

**Keywords:** Tim-3, N-glycosylation, Tim-3L, CD4^+^CD25^+^ T cells

## Abstract

**Background:**

T cell immunoglobulin and mucin domain containing 3 protein (Tim-3) expressed on terminally differentiated Th1 cells plays a suppressive role in Th1-mediated immune responses. Recently, it has been shown that N-glycosylation affects the binding activity of the Tim-3-Ig fusion protein to its ligand, galectin-9, but the binding properties of non-glycosylated Tim-3 on CD4^+^CD25^+^ T cells has not been fully examined. In this study, we produced recombinant Tim-3-Ig fusion proteins in different cellular sources and its N-glycosylation mutant forms to evaluate their binding activities to CD4^+^CD25^+^ T cells.

**Methods:**

We isolated and cloned Tim-3 cDNA from BALB/C mouse splenocytes. Then, we constructed a mammalian expression vector and a prokaryotic expression vector for the Tim-3-Ig fusion protein. Using a site directed mutagenesis method, plasmid vectors for Tim-3-Ig N-glycosylation mutant expression were produced. The recombinant protein was purified by protein A sepharose column chromatography. The binding activity of Tim-3-Ig fusion protein to CD4^+^CD25^+^ T cells was analyzed using flow cytometry.

**Results:**

We found that the nonglycosylated Tim-3-Ig fusion proteins expressed in bacteria bound to CD4^+^CD25^+^ T cells similarly to the glycosylated Tim-3-Ig protein produced in CHO cells. Further, three N-glycosylation mutant forms (N53Q, N100Q, N53/100Q) of Tim-3-Ig showed similar binding activities to those of wild type glycosylated Tim-3-Ig.

**Conclusion:**

Our results suggest that N-glycosylation of Tim-3 may not affect its binding activity to ligands expressed on CD4^+^CD25^+^ T cells.

## INTRODUCTION

T cell immunoglobulin and mucin domain containing 3 protein (Tim-3) was identified using monoclonal antibodies specific to TH1-cell clones (1). Tim-3 is expressed on the surface of differentiated TH1 cells, CD8 T cells and some macrophages (2). Also, Tim-3 expression in mast cells and NK cells has been recently demonstrated (2,3). Tim-3 belongs to the TIM family of genes because it contains an immunoglobulin (Ig) V-like domain and a mucin-like domain (4).

The function of Tim-3 has been revealed using antibodies against Tim-3 and Tim-3-Ig fusion proteins. Administration of Tim-3 blocking antibody aggravates experimental autoimmune encephalitis (1). Injection of Tim-3-Ig into mice induces hyperproliferation of TH1 cells as well as TH1 cytokine release and abrogated tolerance induction in TH1 cells (2). Blocking antibody against Tim-3 accelerated autoimmune diabetes partly due to the hindrance of the function of CD4^+^CD25^+^ regulatory T cells (5). Further, injection of Tim-3 antibody into coxsackievirus-infected mice exacerbates cardiac inflammation and reduces expression of CTLA-4 on T cells and B7.1 on APCs (6). The treatment with Tim-3 antibody enhanced interferon-γ expression and cytotoxicity by donor CD8+ T cells against host alloantigen, resulting in accelerated acute graft versus host disease in mice (7).

As a Tim-3 ligand, galectin-9 was identified from a CD8 T cell clone that showed a Tim-3 binding profile on flow cytometric analysis (8). Interaction of galectin-9 with Tim-3 requires carbohydrate motif(s) on Tim-3IgV (8). A soluble isoform (sTim-3-Ig), containing the IgV domain but lacking the mucin domain binds to galectin-9 (8). Further, in coprecipitation experiments with untreated or *N*-glycosidase F-treated (deglycosylated) sTim-3-Ig and galectin-9, only untreated sTim-3-Ig was able to precipitate galectin-9, implying that the carbohydrates on the sTim-3-Ig bind to galectin-9 (8). N-glycosidase F removes unaltered most of the common N-linked carbohydrates from proteins while hydrolyzing the originally glycosylated Asn residue to Asp (9).

N-glycosylation consensus sequence, Asn-Xaa-Ser/Thr, occurrs twice in Tim-3 IgV like domain. Even before the identification of galectin-9 as a Tim-3 ligand, the importance of IgV domain for ligand binding was revealed by demonstrating putative ligand(s) expression of CD4^+^CD25^+^ T cells using recombinant Tim-3 IgV protein (10). Although galectin-9 expression in regulatory T cells has not yet been demonstrated, *in vivo* administration of galectin-9 leads selective TH1 cell deletion and promotion of regulatory T cell induction (11). Despite the importance of N-linked glycosylation of the IgV domain of Tim-3 in its ligand binding activity, it has not been directly examined whether glycosylation of these sequences is involved in binding to ligands expressed on CD4^+^CD25^+^ T cells. In this study, we produced recombinant Tim-3-Ig fusion proteins in different cellular sources and its N-glycosylation mutant forms. We evaluated their binding activities to CD4^+^CD25^+^ T cells.

## MATERIALS AND METHODS

### Construction of plasmids for Tim-3-Ig fusion protein expression

The Tim-3 gene was cloned from splenocytes of a BALB/c mouse. First, splenocytes (1×10^6^) were stimulated with Concanavalin A (1 ug/ml) for 2 days, and then, total RNA was extracted by RNA-Bee RNA isolation reagent (Tel-Test, Friendswood, TX, USA). Total RNA was subjected to reverse transcription using RNase H- reverse transcriptase (Invitrogen, Carsbade, CA, USA). The cDNA was amplified using Tim-3 specific primers (2). The PCR products were cloned into pCR2.1-TOPO vector (Invitrogen) and sequenced. Tim-3 gene sequences were registered in NCBI database. The gene encoding the extracellular domain of Tim-3 was amplified with specific primers (Tim-3-Forward (*Nhe*I)): GCTAGCATGTTTTCAGGTCTTACCCTCAACTGTG and Tim-3-Reverse (*Bgl*II)): AGATCTTCTGATCGTTTCTCCAGAGTCCTTAATTTCATCAG), and the PCR product was inserted into pIRES2-EGFP vector (Clontech) using the *Nhe*I and *Bgl*II site. The gene encoding IgG1 heavy chain CH2CH3 was amplified using pTOPO-hIgG1 vector (kind gift of Dr. Kwon, Ajou University, Korea) and the specific primers (HIgC-Foward(*Bgl*II)): GAAGATCTGCACCTGAACTCCTGGGG and HIgC-Reverse(*Bam*HI)): CGGGATCCTCATTTACCCTGCGACAG). Using the *Bgl*II and *Eco*RI site, the PCR product was ligated to pIRES2-EGFP downstream of Tim-3 gene. To construct prokaryotic Tim-3-Ig expression vector, Tim-3-Ig DNA fragment was subcloned into pIg20 vector using *Xma*I and *Nco*I. To produce expression vectors for N-glycosylation mutant Tim-3-Ig, the Arg53 and 100Arg of Tim-3 were replaced to glutamine using site directed mutagenesis kit (Clontech) and pIRES-EGFP-Tim-3-Ig. The nucleotide sequences were verified.

### Purification of Tim-3-Ig

In order to produce CD4^+^ Tim-3-Ig protein, Chinese hamster ovary (CHO) cells were transfected with pIRES2-EGFP-Tim-3-Ig vector using Polyethylenimine (PEI, Polyscience, PA USA). After 2 days, the culture supernatant was harvested and Tim-3-Ig was purified using protein A sepharose column (Amersham Biosciences, Uppsala, Sweden). The purity of Tim-3-Ig was examined by SDS-PAGE and by Western blot using peroxidase conjugated anti-human Ig antibody and ECL detection system.

For the production of prokaryotic Tim-3-Ig protein, pIg20-Tim-3-Ig was transformed into BL21 *Escherichia coli* (Invitrogen). The bacteria were allowed to grow in Luria broth media until an OD 600 of 0.8 was achieved. Expression of Tim-3-Ig was induced with addition of 1mM isopropyl-β-D (-)-thiogalactopyranoside (IPTG). Eight hours later the culture supernatant was harvested and Tim-3-Ig was purified.

### Preparation of enriched mouse CD4^+^ T cells

For enrichment of CD4^+^ T cells, spleen and lymph nodes from 6~8-week old mice were harvested and single cell suspensions were prepared. Lymphocytes were enriched by Ficoll-Paque gradient centrifugation at 2,000 rpm for 20 min. CD4^+^ T cells were enriched by positive selection with the CD4^+^ T cell isolation kit (Miltenyi Biotec, Gladbach, Germany).

### FACS analysis for binding activity of Tim-3-Ig protein

The enriched CD4^+^ T cells were incubated with Tim-3-Ig, FITC conjugated anti-CD4 mAb (BD Pharmingen) and PE conjugated anti-CD25 mAb (BD Pharmingen) at 4℃ for 30 min. After incubation, enriched CD4^+^ T cells were washed in PBS containing 2% BSA and then labeled with biotin-conjugated anti-human IgG Ab (BD biosciences) and Streptavidin-PerCP (BD biosciences). Stained cells were analyzed on a BD Vantage system (Becton-Dickinson Co.).

## RESULTS

### Strain differences in Tim-3 amino acid sequences

**Figure 1:**
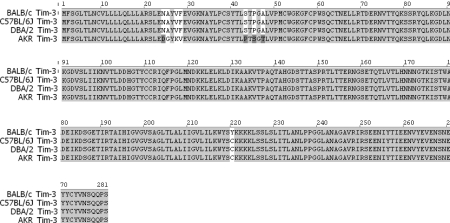
Comparison of Tim-3 aminoacid sequences of BALB/c with three different mouse strains. cDNA of Tim-3 was cloned from activated splenocytes of BALB/c mouse, nucleotide sequences were analyzed and deduced amino acid sequences were shown. Accession numbers of BALB/c Tim-3 are AY553334.1 for nucleotide sequences and AAS5931.1 for amino acid sequences. Accession numbers of C57BL/6, DBA/2 and AKR Tim-3 are BAE28574.1, AAL35776.1 and AAL65156.1, respectively.

First, cDNA of Tim-3 was cloned from activated splenocytes of BALB/c mouse and nucleotide sequences were analyzed. Then, Tim-3 amino acid sequences of BALB/c mouse were compared with those of C57BL/6 (gene bank accesion number: BAE28574.1), DBA/2 (AAL35776.1) and AKR (AAL65156. 1) mice registered in gene bank (Fig. 1). In comparison with all other mouse genotypes, there was only one difference found at the 220th amino acid position in the linear sequence in Tim-3 of BALB/c. Compared with AKR, eight distinct amino acids at the 24th, 25th, 27th, 28th, 43rd, 45th, 47th and 220th sites in the linear amino acid sequence were observed in Tim-3 of BALB/c.

### Tim-3-Ig fusion proteins purified from mammalian and bacterial cells

**Figure 2:**
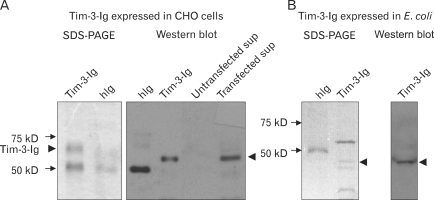
Analysis of Tim-3-Ig purified from CHO cell and *E. coli* culture. (A) Tim-3-Ig was purified from culture supernatant of CHO cells transfected with pIRES2-EGFP-Tim-3-Ig using protein A affinity column chromatography and subjected to SDS-PAGE and Western blot using peroxidase conjugated anti-human IgG antibody and ECL detection system. (B) Tim-3-Ig was purified from culture supernatant of *E. coli* transformed with pIg20-Tim-3-Ig and subjected to SDS-PAGE and Western blot using peroxidase conjugated anti-human IgG antibody and ECL detection system.

The cDNA fragment of Tim-3 extracellular domain and human IgG heavy chain constant region were inserted into a eukaryotic expression vector for the production of Tim-3-Ig fusion protein. CHO cells were transfected with eukaryotic Tim-3-Ig expression plasmid and then the Tim-3-Ig protein was purified from the culture supernatant by affinity column chromatography. Similarly, Tim-3-Ig was purified from *E. coli* transformed with prokaryotic Tim-3-Ig expression vector. Purity of Tim-3-Ig produced in each cell culture type was examined by SDS-PAGE analysis and Western blot (Fig. 2). Due to the glycosylation of Tim-3, the band for Tim-3-Ig purified from CHO cells appeared above the 50 kD standard marker (Fig. 2A). Identification of this band as Tim-3-Ig was performed by Western blot using anti-human IgG Ab reacting with fusion partner. A band of approximate 50 kD protein seen in the Tim-3-Ig lane expressed in CHO cells on SDSPAGE appeared to be fetal bovine serum Ig heavy chain (Fig. 2A). Whereas Tim-3-Ig expressed in *E. coli* was seen as approximate a 45 kD band in the Western blot due to the lack of glycosylation (Fig. 2B). Tim-3-Ig expressed in *E. coli* was partially purified based on SDS-PAGE analysis (Fig. 2B). These results indicated that even though it was partially purified, Tim-3-Ig could be used for testing its binding reactivity to the ligands expressed on CD4^+^ CD25^+^ T cells since its ligand binding activity would be revealed with anti-human IgG Ab reactive to the fusion partner.

### Similar binding reactivity of Tim-3-Ig fusion protein to CD4^+^ CD25^+^ T cells regardless of its cellular sources

**Figure 3:**
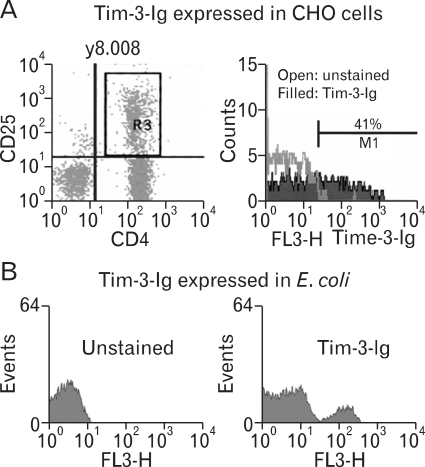
The binding activity of Tim-3-Ig to CD4^+^CD25^+^ T cells. Mouse CD4^+^ T cells were labeled with FITC conjugated anti-CD4 Ab, PE-conjugated anti-CD25 Ab and Tim-3-Ig purified either from CHO cells (A) or from *E. coli* (B). Subsequently, cells were stained with biotin-conjugated anti-human IgG Ab and Streptavidin-PerCP. Cells were gated on CD4 CD25 expression and the binding activity of Tim-3-Ig to these cells was analyzed by flow cytometry.

CD4^+^ CD25^+^ T cells have been reported to express Tim-3 ligand (5). To analyze ligand binding activity of Tim-3-Ig expressed in different cellular sources, enriched murine CD4^+^ T cells were incubated with Tim-3-Ig and subsequently, with anti-human IgG Ab conjugated with immunofluorescence (Fig. 3). Enriched CD4^+^ T cells were gated on CD4^+^ CD25^+^ expression (Fig. 3A left panel) and the binding activity of Tim-3-Ig to these cells was analyzed by flow cytometry. Flow cytometric analysis showed that Tim-3-Ig expressed in CHO cells bound 41% to CD4^+^ CD25^+^ T cells (Fig. 3A right panel). Further, Tim-3-Ig expressed in *E. coli* also bound to CD4^+^ CD25^+^ T cells (Fig. 3B). It should be mentioned that the molecular concentration of Tim-3-Ig used in Fig. 3A was not same as that used in Fig. 3B. These results suggest that glycosylation may not be critical to the binding activity of Tim-3-Ig to the ligand on CD4^+^ CD25^+^ T cells.

### Production of N-glycosylation mutant forms of Tim-3-Ig

**Figure 4:**
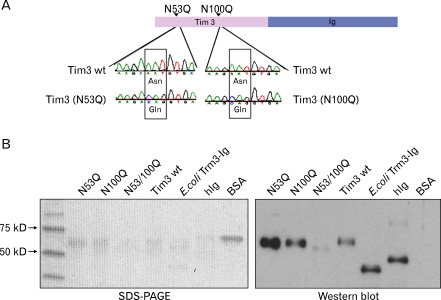
Production of Tim-3-Ig N-glycosylation mutants. (A) Schematic representation and sequence analysis of Tim-3-Ig N-glycosylation mutants, N53Q and N100Q. (B) Tim-3-Ig N-glycosylation mutants were purified from CHO cell culture using affinity column chromatography and subjected to SDS-PAGE analysis and Western blot using peroxidase conjugated anti-human Ig antibody and ECL detection system.

The mucin domain of Tim-3 is not involved in ligand binding activity (2,5). N-glycosylation consensus sequence, Asn-Xaa-Ser/Thr, occurrs twice in Tim-3 IgV like domain. To further confirm the role of Tim-3 glycosylation in ligand binding activity, mutants of two potential N-glycosylation sites of Tim-3 IgV like domain were produced. Nucleotide sequences of eukaryotic expression vectors for N53Q, N100Q, and double mutant N53/100Q were confirmed respectively (Fig. 4A). Then, these mutant forms expressed in CHO cells were purified and confirmed by SDS-PAGE and Western blot (Fig. 4B). Compared with wild type Tim-3-Ig, the molecular weight of the double mutant N53/100Q was significantly smaller. These results indirectly showed the absence of N-glycosylation on these mutants.

### Similar binding reactivity of Tim-3-Ig N-glycosylation mutants to CD4^+^ CD25^+^ T cells

**Figure 5:**
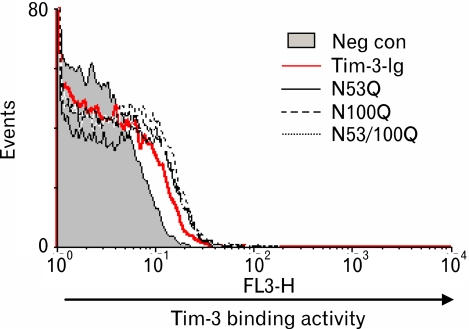
The binding activity of Tim-3-Ig N-glycosylation mutants to CD4^+^CD25^+^ T cells. Mouse CD4^+^ T cells were labeled with FITC conjugated anti-CD4 Ab, PE-conjugated anti-CD25 Ab and either Tim-3-Ig wild type or Tim-3-Ig N-glycosylation mutants. Subsequently, these cells were incubated with biotin-conjugated anti-human IgG Ab and Streptavidin-PerCP. Cells were gated on CD4 CD25 expression and the binding activity of Tim-3-Ig to these cells was analyzed by flow cytometry.

Next the ligand binding activity of N-glycosylation mutant forms was examined (Fig. 5). The concentration of each Tim-3-Ig form was adjusted to 0.75 µM. Since the concentration of Tim-3-Ig was much lower than that used in Fig. 3A, the binding activity of wild Tim-3-Ig was less evident. However, as expected, there was no difference in the binding activity between any of the Tim-3-Ig N-glycosylation mutant forms and wild type Tim-3-Ig implying that N-glycosylation of Tim-3 IgV like domain may not affect ligand binding activity of Tim-3.

## DISCUSSION

Although the studies regarding immunomodulatory function of Tim-3 have been accumulated, the interaction of Tim-3 with its ligand has not been well studied. In this study, the binding activity of Tim-3-Ig fusion protein expressed either in CHO cells or in *E. coli* to its ligand-expressing CD4^+^ CD25^+^ T cells was evaluated, and the role of N-glycosylation of Tim-3 in its ligand interaction was investigated.

Supporting previous reports (2), Tim-3-Ig expressed in CHO cells showed binding activity to CD4^+^ CD25^+^ T cells. Also, bacterially expressed Tim-3-Ig bound to these cells. Like our results, a recent report by Cao et al. shows bacterially expressed Tim-3 IgV tetramer bound to Foxp3^+^ T cells and suggests a second ligand for Tim-3 other than galectin-9 (10). In line with these findings, N-glycosylation mutants of Tim-3-Ig bound CD4^+^ CD25^+^ T cells comparable to the wild type Tim-3-Ig. Our results suggest that glycosylation may not be required for the interaction of Tim-3 with its ligands on CD4^+^ CD25^+^ T cells.

Although bacterially expressed Tim-3-Ig showed binding activity to CD4^+^ CD25^+^ T cells, it should be addressed whether its biological activity is same as Tim-3-Ig expressed in mammalian cells. The reasons are as follows. First, galectin-9, a known Tim-3 ligand, requires glycosylation of Tim-3 for its binding (8). Second, until now Tim-3-Ig expressed in eukaryotic cells has been used in functional studies. Third, the stabilities of bacterially expressed Tim-3-Ig and Tim-3-Ig expressed in eukaryotic cells have not been compared. Glycosylation may confer stability on Tim-3-Ig.

For the therapeutic use of TIM-3-Ig, convenient massive production of TIM-3-Ig will be desirable. In spite of the general advantages of bacterial expression systems such as low cost, our bacterial expression system revealed critical disadvantages in the purification step. Even though we tried cation exchange column chromatography, the purity did not greatly improved (data not shown). In terms of purity, the mammalian expression system was better for Tim-3-Ig production.

In this study, BALB/c Tim-3 cDNA and deduced amino acid sequences were identified. Compared with Tim-3 of other murine genotypes, BALB/c Tim-3 differs at 220th amino acid position. BALB/c Tim-3 showed 8 amino acid differences compared to AKR Tim-3. It may be interesting to study whether these differences have an effect on the function of Tim-3.

To conclude, our results suggest that Tim-3-Ig fusion protein may bind to CD4^+^ CD25^+^ T cells without need to be glcosylated including N-glycosylation of IgV domain. The contribution of glycosylation to the stability and function of Tim-3 remains to be revealed.

## References

[1] Monney L, Sabatos CA, Gaglia JL, Ryu A, Waldner H, Chernova T, Manning S, Greenfield EA, Coyle AJ, Sobel RA, Freeman GJ, Kuchroo VK (2002). Th1-specific cell surface protein Tim-3 regulates macrophage activation and severity of an autoimmune disease. Nature.

[2] Sabatos CA, Chakravarti S, Cha E, Schubart A, Sanchez-Fueyo A, Zheng XX, Coyle AJ, Strom TB, Freeman GJ, Kuchroo VK (2003). Interaction of Tim-3 and Tim-3 ligand regulates T helper type 1 responses and induction of peripheral tolerance. Nat Immunol.

[3] Wiener Z, Kohalmi B, Pocza P, Jeager J, Tolgyesi G, Toth S, Gorbe E, Papp Z, Falus A (2007). TIM-3 is expressed in melanoma cells and is upregulated in TGF-beta stimulated mast cells. J Invest Dermatol.

[4] Kuchroo VK, Meyers JH, Umetsu DT, DeKruyff RH (2006). TIM family of genes in immunity and tolerance. Adv Immunol.

[5] Sanchez-Fueyo A, Tian J, Picarella D, Domenig C, Zheng XX, Sabatos CA, Manlongat N, Bender O, Kamradt T, Kuchroo VK, Gutierrez-Ramos JC, Coyle AJ, Strom TB (2003). Tim-3 inhibits T helper type 1-mediated auto- and alloimmune responses and promotes immunological tolerance. Nat Immunol.

[6] Frisancho-Kiss S, Nyland JF, Davis SE, Barrett MA, Gatewood SJ, Njoku DB, Cihakova D, Silbergeld EK, Rose NR, Fairweather D (2006). Cutting edge: T cell Ig mucin-3 reduces inflammatory heart disease by increasing CTLA-4 during innate immunity. J Immunol.

[7] Oikawa T, Kamimura Y, Akiba H, Yagita H, Okumura K, Takahashi H, Zeniya M, Tajiri H, Azuma M (2006). Preferential involvement of Tim-3 in the regulation of hepatic CD8+ T cells in murine acute graft-versus-host disease. J Immunol.

[8] Zhu C, Anderson AC, Schubart A, Xiong H, Imitola J, Khoury SJ, Zheng XX, Strom TB, Kuchroo VK (2005). The Tim-3 ligand galectin-9 negatively regulates T helper type 1 immunity. Nat Immunol.

[9] Medzihradszky KF (2008). Characterization of site-specific N-glycosylation. Methods Mol Biol.

[10] Cao E, Zang X, Ramagopal UA, Mukhopadhaya A, Fedorov A, Fedorov E, Zencheck WD, Lary JW, Cole JL, Deng H, Xiao H, Dilorenzo TP, Allison JP, Nathenson SG, Almo SC (2007). T cell immunoglobulin mucin-3 crystal structure reveals a galectin-9-independent ligand-binding surface. Immunity.

[11] Seki M, Oomizu S, Sakata KM, Sakata A, Arikawa T, Watanabe K, Ito K, Takeshita K, Niki T, Saita N, Nishi N, Yamauchi A, Katoh S, Matsukawa A, Kuchroo V, Hirashima M (2008). Galectin-9 suppresses the generation of Th17, promotes the induction of regulatory T cells, and regulates experimental autoimmune arthritis. Clin Immunol.

